# Cerebral Metabolic Analysis of Patients With Colorectal Cancer and Chronic Enteritis: Inquiry Into Gut-Brain Crosstalk

**DOI:** 10.3389/fnins.2022.822891

**Published:** 2022-02-25

**Authors:** Jie Ma, Jia-Jia Wu, Xiang-Xin Xing, Bei-Bei Huo, Xin Gao, Zhen-Zhen Ma, Si-Si Li, Mou-Xiong Zheng, Xu-Yun Hua, Jian-Guang Xu

**Affiliations:** ^1^School of Rehabilitation Science, Shanghai University of Traditional Chinese Medicine, Shanghai, China; ^2^Center of Rehabilitation Medicine, Yueyang Hospital of Integrated Traditional Chinese and Western Medicine, Shanghai University of Traditional Chinese Medicine, Shanghai, China; ^3^Universal Medical Imaging Diagnostic Center, Shanghai, China; ^4^Department of Rehabilitation Medicine, Longhua Hospital, Shanghai University of Traditional Chinese Medicine, Shanghai, China; ^5^Department of Traumatology and Orthopedics, Yueyang Hospital of Integrated Traditional Chinese and Western Medicine, Shanghai University of Traditional Chinese Medicine, Shanghai, China; ^6^Engineering Research Center of Traditional Chinese Medicine Intelligent Rehabilitation, Ministry of Education, Shanghai, China

**Keywords:** cerebral metabolism, colorectal cancer, chronic enteritis, SSM/PCA, metabolic connectivity

## Abstract

Gut-brain crosstalk has been demonstrated previously. However, brain metabolic patterns of colorectal cancer and chronic enteritis remain unclear. A better understanding of gut-brain crosstalk from a radiological perspective is necessary. We conducted a retrospective study in which we acquired ^18^F-fluorodeoxyglucose positron emission tomography in 45 colorectal cancer cases, 45 age- and sex-matched chronic enteritis patients, and 45 age- and sex-matched healthy controls. We calculated a scaled sub-profile pattern based on principal component analysis and metabolic connectivity to explore the brain metabolic model and analyzed correlations between various brain regions and cancer to identify potential neuroimaging markers for non-pharmaceutical therapies. We found a characteristic cerebral metabolic pattern in colorectal cancer patients, which mainly involved visceral sensation and both affective and cognitive psychological processes. The metabolic patterns of patients with colorectal cancer and chronic enteritis were similar but not identical. The metabolic connectivity of the postcentral gyrus and paracentral lobule was found to be significantly different between the controls and patients with colorectal cancer (*p* < 0.05, false discovery rate correction). The maximal standard uptake value of the cancer focus in colorectal cancer patients was negatively correlated with the dorsolateral superior frontal gyrus (*p* < 0.05). Patients with colorectal cancer may show abnormal glucose cerebral metabolism characterized by “point-line-surface.” This preliminary study revealed the cerebral metabolic characteristics and neurobiological mechanisms of colorectal cancer and chronic enteritis (ChiCTR2000041020; registered December 16, 2020).

## Introduction

The association between the gut and brain in the etiology, pathogenesis, and pathophysiology of disease has been investigated extensively. Technological progress has provided valuable insight into the complex and close relationship between the gut and brain (i.e., the gut-brain axis) in both healthy and diseased states. The gut-brain axis is the bidirectional communication between the gut and the brain that maintains gastrointestinal homeostasis and affects higher cognitive functions, emotion, and motivation *via* neural pathways and cellular and humoral mediators (De Palma et al., 2014). Numerous studies on the gut-brain axis have been carried out in both nervous system and intestinal diseases. In recent years, the effects of intestinal disorders related to intestinal microflora in the central nervous system have received increased attention. Major intestinal disorders, namely, inflammatory bowel disease, irritable bowel syndrome, and colorectal cancer (CRC), are associated with intestinal microflora (De Palma et al., 2014).

Advanced neuroimaging [e.g., functional magnetic resonance imaging and positron emission tomography (PET)] can be used to reveal functional damage and understand brain function in intestinal diseases. Converging evidence from brain imaging studies suggests that the change of neural plasticity involved in cognition, emotion, and visceral sensation was found in inflammatory bowel disease (Gray et al., 2018) and irritable bowel syndrome (Hong et al., 2014). However, to date, only one brain imaging study has investigated alterations of cerebral neural activity in CRC patients with depressive symptoms, which revealed a spontaneous decrease in neural activity in the occipital and parietal lobes (Zhao et al., 2019). CRC patients often experience visceral pain, insomnia, and emotional distress (Holch et al., 2011), especially depression, which are significant factors for brain imaging changes; moreover, these symptoms seriously affect patients’ quality of life (Berluti et al., 2020). Furthermore, visceral pain, insomnia, and emotional distress influence each other. In addition, several studies have shown cerebral metabolic abnormalities in cancer patients, and this change varies depending on the type of cancer (Li et al., 2015). However, there has not been any study comparing brain function patterns between patients with enteritis and colon cancer. Inflammation is closely related to tumors and plays an important role in tumor occurrence, tumor development, and prognosis (McSorley et al., 2018). However, the pathological process of inflammation differs from that of tumors. Inflammation is a process of “destruction-regeneration-repair,” whereas a tumor undergoes a process of “destruction-regeneration-destruction.” Therefore, we assume that CRC patients may exhibit distinct and complex cerebral metabolic abnormalities, and the effects of CRC and enteritis on brain plasticity likely have both similarities and differences.

The brain relies primarily on glucose as its primary energy source under normal physiological conditions. Glucose nourishes tissues, provides information for the central regulatory circuits for metabolism and feeding conditions, and is predominantly used in neurons to maintain an antioxidant status (Magistretti and Allaman, 2015). PET imaging provides regional information and reflects the functional diversity of highly specialized brain regions. Therefore, PET is particularly suited to the study of cerebral metabolism and structure (Raichle and Mintun, 2006). Thus, we conducted a retrospective study in patients with CRC and healthy controls by analyzing ^18^F-fluorodeoxyglucose positron emission tomography (^18^F-FDG-PET) data to explore the brain metabolic model of CRC. We also studied the cerebral metabolic characteristics of chronic enteritis, which is considered one of the common precancerous lesions, to confirm that cerebral metabolic mapping of CRC differs from that of other intestinal diseases. Disease-specific cerebral metabolic characteristics of CRC and chronic enteritis were identified using a multivariate spatial covariance technique known as scaled sub-profile model based on principal component analysis (SSM/PCA) applied to ^18^F-FDG-PET images (Tomše et al., 2017). The connections between and within the cerebral cortex are the basis for the operation of local cortical circuits, which are an essential factor for whole-brain function, and various brain disorders have been shown to present with network abnormalities (Haider et al., 2006). Therefore, we also calculated the metabolic connectivity between brain regions. Additionally, we analyzed the correlation between brain regions and tumors to identify potential neuroimaging markers for non-pharmaceutical therapies. This preliminary study aimed to reveal the cerebral metabolic characteristics and brain biological mechanisms of CRC and chronic enteritis.

## Materials and Methods

### Study Subjects

The sample used in this retrospective study comprised 45 CRC cases, 45 age- and sex-matched chronic enteritis patients, and 45 age- and sex-matched healthy controls from Shanghai, China. The CRC group fulfilled the following inclusion criteria: (1) aged ≥ 18 years; (2) diagnosis of CRC confirmed by histopathological examination and ^18^F-FDG-PET; (3) did not undergo treatment before ^18^F-FDG-PET; and (4) no metastasis. The chronic enteritis group fulfilled the following inclusion criteria: (1) aged ≥ 18 years; (2) diagnosis of chronic enteritis confirmed by clinical manifestations of recurrent abdominal pain, diarrhea, and other symptoms, endoscope examination, and ^18^F-FDG-PET; (3) did not undergo treatment in the previous month; and (4) course of the disease is >1 year. The control group fulfilled the following inclusion criteria: (1) aged ≥ 18 years; (2) no symptoms of the digestive system, such as abdominal pain and diarrhea; and (3) no abnormal FDG metabolism in the brain or intestine in ^18^F-FDG-PET. Diagnoses were made independently by three senior radiology experts, according to their experience combined with Siemens PET data postprocessing software (MI nerve). Exclusion criteria for all subjects were: (1) neurological diseases (e.g., stroke, dementia, Parkinson’s disease, space-occupying lesions, and epilepsy); (2) severe systemic diseases (e.g., other cancer, severe abnormal glucose metabolism, serious heart, liver, kidney, blood system diseases, or infectious diseases); and (3) psychiatric diseases (e.g., anxiety, depression, and schizophrenia). All cases underwent whole-body ^18^F-FDG-PET. This retrospective study was approved by the institutional review board of Yueyang Hospital (No. 2020-188).

### Image Acquisition

Corresponding PET images of subjects were acquired on a Biograph mMR scanner (Siemens, Munich, Germany). Subjects fasted for at least 6 h before undergoing PET imaging, and blood glucose levels were measured to ensure the absence of hyperglycemia (>150 mg/dL) before each scan. Subjects were required to close their eyes and keep calm throughout the examination. The acquisition time of the PET scan was approximately 40–50 min, with an injection of 3.7 MBq/kg ^18^F-FDG. PET datasets were acquired using three-dimensional (3D) image reconstruction with ordered subset expectation maximization with 3 iterations, 21 subsets, and a Gaussian filter at 4.0 mm full width at half maximum (slice thickness of 2.03 mm; acquisition matrix 172 × 172; and in-plane resolution 4.17 mm × 4.17 mm).

### Data Processing

**FIGURE 1:**
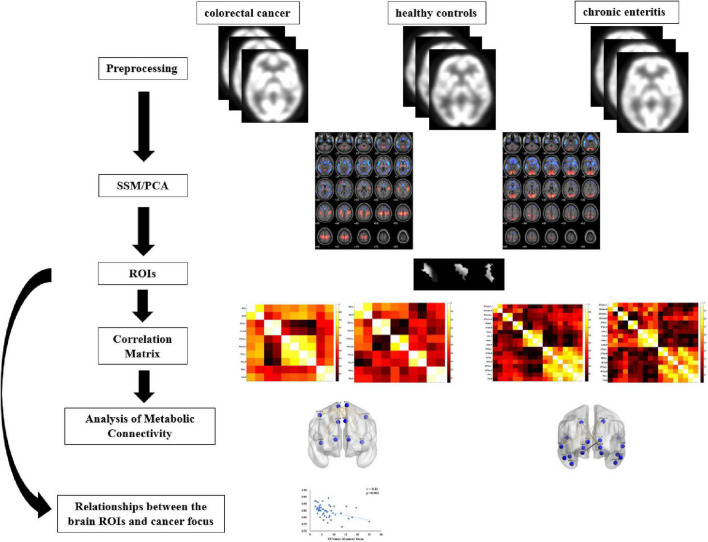
Flowchart of analysis.

Figure 1 shows the pipeline of our framework. The details are described in the following subsections. The ^18^F-FDG-PET brain images of each subject were preprocessed and processed using the ScAnVP Software 7.0^1^ and the Statistical Parametric Mapping (SPM) 12.0^2^, which is based on the Matlab 2013b platform. Preprocessing was performed as follows: (1) PET brain images were converted into Neuroimaging Informatics Technology Initiative or Analyze format (SSM/PCA); (2) origin was set to the anterior commissure; (3) PET images were spatially normalized using PET template in the standard Montreal Neurological Institute (MNI); and (4) spatial smoothing was applied using a Gaussian kernel of 8-mm full width at half maximum.

### Voxel-Based Scaled Sub-Profile Model/Principal Component Analysis

After preprocessing PET data, the spatially normalized and smoothed images were thresholded uniformly to limit the analysis to gray matter areas (the fraction of the volume max: default = 0.35 for PET). The data were then log-transformed, and each subject-dependent row was centered by subtracting the row mean of the log values. The resulting subject residual profile images represented deviations from both the subject and group means. Finally, PCA was applied to the covariance matrix of the double-centered matrix to derive orthogonal eigenvectors, associated eigenvalues, and subject scores. The spatial eigenvectors were principal component (PC) image patterns of the SSM/PCA analysis.

### Analysis Based on Regions of Interest

The regions of interest (ROIs) were selected according to the PC image patterns of the SSM/PCA analysis, including 10 brain regions of CRC and 12 brain regions of chronic enteritis (cluster size > 100; Table 1) and extracted from PET 3D imaging data using predefined automated anatomical labeling atlas with 90 volumes-of-interest (VOIs; AAL-90) (Tzourio-Mazoyer et al., 2002). The average standard uptake value (SUV) of each ROI was extracted for analysis. In addition, in each subject, the mean ^18^F-FDG uptake in the ROIs was regressed by the mean ^18^F-FDG uptake of the whole brain to correct for the variability in injected activity (Huo et al., 2019).

**TABLE 1:** Region of the principal component image patterns in the scaled sub-profile model based on principal component analysis and abbreviations.

Colorectal cancer		Chronic enteritis	
Region name	Abbreviation	Region name	Abbreviation
Superior frontal gyrus, dorsolateral	SFGdor	Superior frontal gyrus, dorsolateral	SFGdor
Insula	INS	Middle frontal gyrus, orbital part	MFGorb
Median cingulate and paracingulate gyri	DCG	Inferior frontal gyrus, triangular part	IFGtri
Postcentral gyrus	PoCG	Calcarine fissure and surrounding cortex	CAL
Precuneus	PCUN	Lingual gyrus	LING
Paracentral lobule	PCL	Temporal pole: superior temporal gyrus	STGp
Thalamus	THA	Middle temporal gyrus	MTG
Middle temporal gyrus	MTG	Temporal pole: middle temporal gyrus	MTGp
Inferior temporal gyrus	ITG	Inferior temporal gyrus	ITG
Calcarine fissure and surrounding cortex	CAL	Caudate nucleus	CAU
		Paracentral lobule	PCL
		Median cingulate and paracingulate gyri	DCG

### Construction and Analysis of Metabolic Connectivity

We found that the above brain regions were mainly related to cognitive function and visceral sensation. Thus, we analyzed metabolic connections in brain regions related to psychological processes (both affective and cognitive) and visceral sensation in patients with CRC and those of brain regions related to psychological processes in patients with chronic enteritis.

The method for the construction and analysis of metabolic connectivity was based on the ROIs. The nodes of the ROIs and the edges between two ROIs were used to describe significant metabolic relationships between two ROIs. Pearson’s correlation coefficients (*r*) between each pair of ROIs were calculated in an inter-subject manner, and a correlation matrix was obtained for each group. A weighted undirected network matrix was constructed for patients and controls, where the strength of each connection was defined by the correlation coefficients.

### Statistical Analysis

SPSS (version 21.0; IBM, Armonk, NY, United States) was used to analyze the clinical data. Data are expressed as means ± standard deviations for continuous variables and number (%) for categorical variables. For the demographic data, one-way analysis of variance was used for continuous variables, and a χ*^2^* test was used for categorical variables. Only clinical data findings with a two-tailed *p* < 0.05 were considered significant. For the evaluation of discriminating PCs (Tomše et al., 2017; Meles et al., 2018), we conducted *f*-tests and two-sample *t*-tests with equal or unequal variances (*p* < 0.05). The total variance of PCs to be examined needed to be >50%, and the variance of individual PCs needed to be >8% to avoid noise. If PC*_*i*_* met these criteria, then project_group invariant subprofile00*i*.img (project_gis00*i*.img) was considered the SSM/PCA pattern. We performed a permutation test on all possible connections to identify significant differences in metabolic connectivity between patients and controls. Randomly reassigned labels were permuted 10,000 times for each ROI. Only findings with a two-tailed *p* < 0.05 were considered significant for the subject scaling factor (SSF) and metabolic connectivity analyses. False discovery rate (FDR) correction was applied for multiple comparisons. Partial correlation analysis was performed to investigate associations between the average SUVs of specific ROIs and the SUV_max_ of the cancer focus, with covariates of age, blood glucose, and body mass index (BMI). The partial correlation analysis was considered significant at *p* < 0.05.

## Results

### Demographics

The demographic and clinical characteristics of participants are summarized in Table 2. The three groups did not differ significantly in age, sex, BMI, or blood glucose (*p* > 0.05).

**TABLE 2:** Demographic and clinical features of patients and controls.

Variables	Colorectal cancer (*n* = 45)	Chronic enteritis (*n* = 45)	Healthy controls (*n* = 45)	*p*-value
Age (*y*)	53.07 ± 8.29	50.89 ± 8.62	49.73 ± 8.18	0.16^a^
Men (*n*,%)	27(60)	26(58)	30(67)	0.66^b^
BMI (*kg/m*^2^)	22.41 ± 2.63	23.75 ± 6.75	24.10 ± 2.96	0.18^a^
Blood glucose (*mmol/l*)	5.58 ± 1.19	5.74 ± 1.12	5.39 ± 0.90	0.31^a^

*^a^One-way analysis of variance; ^b^Chi-square test.*

### Pattern Identification of Colorectal Cancer and Chronic Enteritis

**FIGURE 2:**
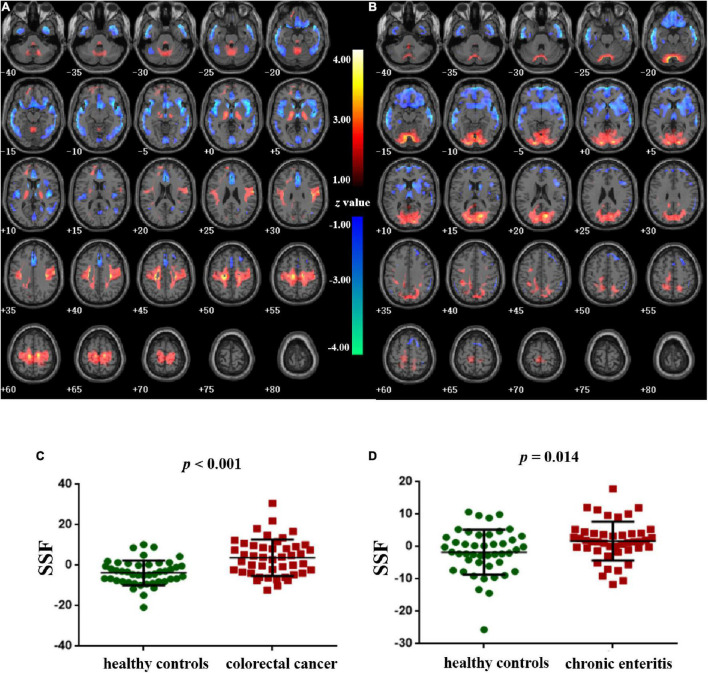
Pattern identification of colorectal cancer and chronic enteritis. **(A)** The *z*-transformed colorectal cancer pattern (with |*z*| > 1) and the subject scaling factor (SSFs) **(C)** of the datasets. **(B)** The *z*-transformed chronic enteritis pattern (with |*z*| > 1) and the SSFs **(D)** of the datasets. Positive and negative *z*-values represent higher and lower metabolism, respectively, in the colorectal cancer/chronic enteritis group than in healthy controls. The *z*-coordinates of each slice are from –40 to 80 mm with a slice spacing of 5 mm.

Figures 2A–D shows the topography of *z*-transformed CRC/chronic enteritis patterns with a threshold of |*z*| > 1 and their SSFs of the CRC and chronic enteritis groups. Combining the SSF distribution and the CRC/chronic enteritis pattern, a positive *z* represented a higher metabolism in the CRC/chronic enteritis group than in the healthy controls. Inversely, a negative *z* represented a lower metabolism in the CRC/chronic enteritis group than in the healthy controls. The central brain regions are shown in Table 3. SSF values were significantly higher in the patient group for each of these patterns than those in the healthy controls.

**TABLE 3:** Main brain regions of colorectal cancer and chronic enteritis patterns.

Brain regions	Cluster size	Cluster centroid MNI coordinates			*z*-value
		*x*	*y*	*z*	
**Colorectal cancer-pattern**					
PoCG.R	1,723	6	−4	20	2.35
PoCG.L	1,436	−58	−10	26	1.46
MTG.R	1,384	−60	−16	−22	–2.70
INS.R	1,202	40	18	−10	–3.58
ITG.L	1,106	−56	−54	−10	–1.97
ITG.R	1,035	60	−22	−24	–2.34
SFGdor.L	559	−14	62	18	2.07
PCL.R	539	8	−36	72	1.90
CAL.L	396	0	−86	−2	–1.92
THA.L	387	−16	−14	2	2.50
DCG.R	288	2	28	30	–2.72
SFGdor.R	166	24	32	46	–1.31
CAL.R	131	26	−52	4	–2.11
PCUN.L	103	−12	−78	30	1.31
**Chronic enteritis-pattern**					
LING.R	1,555	30	−84	−18	1.51
CAL.R	1,328	14	−74	18	2.81
SFGdor.R	1,092	24	28	54	–1.89
ITG.L	983	−58	−18	−26	–3.58
MTG.R	884	64	−22	−8	–3.26
PCL.L	579	−4	−30	74	1.66
CAU.R	480	8	12	4	–2.61
MFGorb.L	460	0	60	−2	–3.44
IFGtri.R	352	44	14	22	–1.76
DCG.L	258	−12	0	40	1.17
STGp.R	180	46	14	−22	–2.57
MTGp.L	107	−34	14	−34	–2.15

*CAL.L, left calcarine fissure and surrounding cortex; CAL.R, right calcarine fissure and surrounding cortex; CAU.R, right caudate nucleus; DCG.L, right median cingulate and paracingulate gyri; DCG.R, right median cingulate and paracingulate gyri; IFGtri.R, right inferior frontal gyrus, triangular part; INS.R, right insula; ITG.L, left inferior temporal gyrus; ITG.R, right inferior temporal gyrus; LING.R, right lingual gyrus; MFGorb.L, left orbital middle frontal gyrus; MTGp.L, left temporal pole: middle temporal gyrus; MTG.R, right middle temporal gyrus; PCL.L, left paracentral lobule; PCUN.L, left precuneus; PCL.R, right paracentral lobule; PoCG.L, left postcentral gyrus; PoCG.R, right postcentral gyrus; SFGdor.L: left dorsolateral superior frontal gyrus; SFGdor.R, right dorsolateral superior frontal gyrus; STGp.R, Temporal pole: right superior temporal gyrus; and THA.L, left thalamus.*

### Direct Comparison of Metabolic Connectivity

**FIGURE 3:**
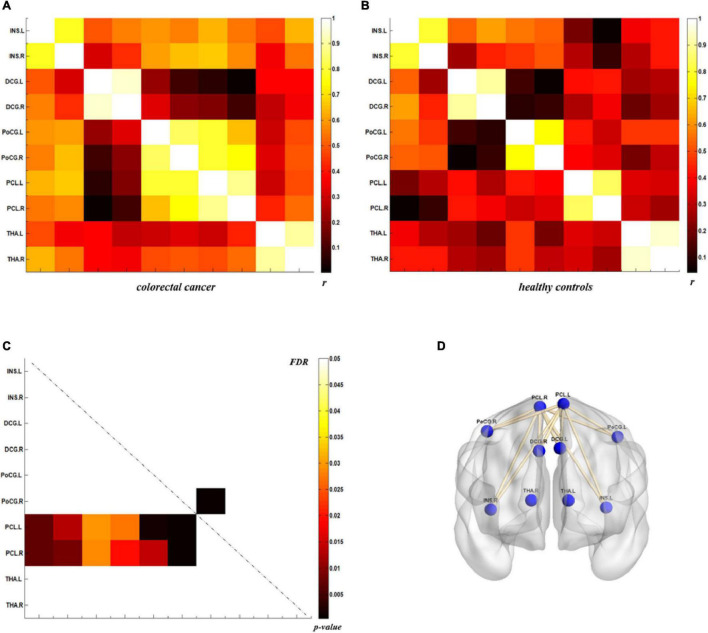
Metabolic connectivity of brain regions related to visceral sensation in the colorectal cancer group and healthy controls. **(A,B)** The *r*-value maps for the colorectal cancer group and healthy controls, respectively. **(C)** To find statistically significant differences in metabolic connectivity between models and controls, we calculated *p*-values for connections using a permutation test (*p* < 0.05). Significant differences in metabolic connectivity after false discovery rate (FDR) correction are represented by the upper triangular matrix, and those without FDR correction are represented by the lower triangular matrix. **(D)** The anatomical distribution of significantly different links between patients with colorectal cancer and healthy controls. *r*: correlation coefficient.

**FIGURE 4:**
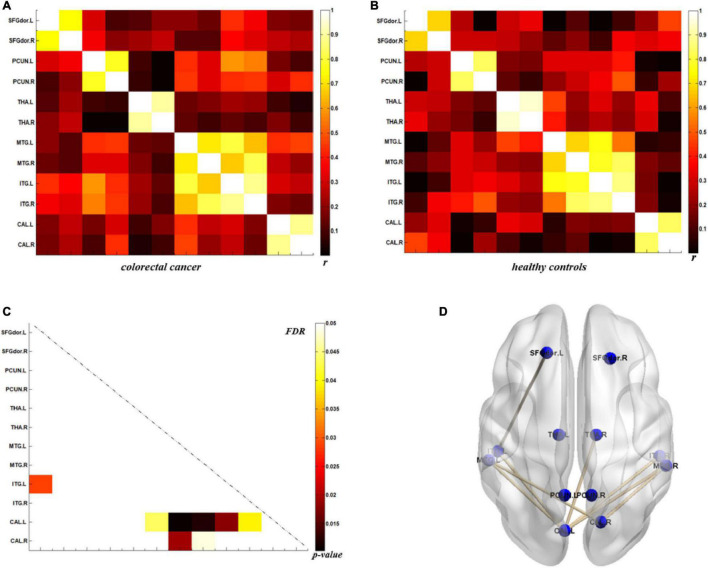
Metabolic connectivity of brain regions related to psychological processes in the colorectal cancer group and healthy controls. **(A,B)** The *r*-value maps for colorectal cancer and healthy controls, respectively. **(C)** To find statistically significant differences in metabolic connectivity between models and controls, we calculated *p*-values for connections using a permutation test (*p* < 0.05). Significance differences in metabolic connectivity after false discovery rate (FDR) correction are represented by the upper triangular matrix, and those without FDR correction are represented by the lower triangular matrix. **(D)** The anatomical distribution of significantly different links between patients with colorectal cancer and healthy controls. *r*: correlation coefficient.

**FIGURE 5:**
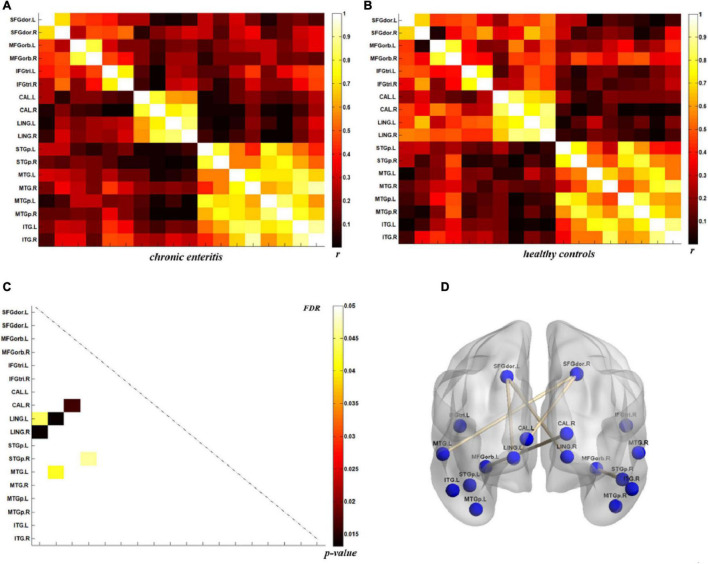
Metabolic connectivity of brain regions related to psychological processes in the chronic enteritis group and healthy controls. **(A,B)** The *r*-value maps for chronic enteritis patients and healthy controls, respectively. **(C)** To find statistically significant differences in metabolic connectivity between models and controls, we calculated *p*-values for connections using a permutation test (*p* < 0.05). Significant differences in metabolic connectivity after false discovery rate (FDR) correction are represented by the upper triangular matrix, and those without FDR correction are represented by the lower triangular matrix. **(D)** The anatomical distribution of significantly different links between patients with chronic enteritis and healthy controls. *r*: correlation coefficient.

The analysis of metabolic connectivity was based on the ROIs, which were chosen according to the PC image patterns from the SSM/PCA analysis. To directly compare the connectivity between controls and CRC/chronic enteritis patients, we performed a permutation test. The CRC group showed significantly different *r*s between the following pairwise VOIs: INS.L-PCL.L, INS.L-PCL.R, INS.R-PCL.L, INS.R-PCL.R, MCC.L-PCL.L, MCC.L-PCL.R, MCC.R-PCL.L, MCC.R-PCL.R, PoCG.L-PCL.L, PoCG.L-PCL.R, PoCG.R-PCL.R, SFGdor.L-ITG.L, CAL.L-THA.R, CAL.L-MTG.L, CAL.L-MTG.R, CAL.L-ITG.L, CAL.L-ITG.R, CAL.R-MTG.L, and CAL.R-MTG.R (*p* < 0.05 uncorrected; Figures 3, 4). Chronic enteritis group showed significantly different *r*s between the following pairwise VOIs: SFGdor.L-LING.L, SFGdor.L-LING.R, SFGdor.R-LING.L, SFGdor.R-MTG.L, MFGorb.L-CAL.R, and MFGorb.R-STGp.R (*p* < 0.05 uncorrected; Figure 5). Only PoCG.R-PCL.L survived FDR correction for the direct comparison of the connectivity between the control and CRC groups (*p* < 0.05).

### Relationships Between the SUV_mean_ of the Brain Region of Interests and the SUV_max_ of the Cancer Focus in Colorectal Cancer Patients

**FIGURE 6:**
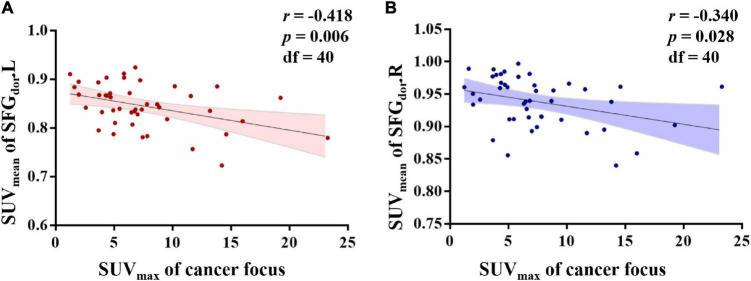
Relationships between the standard uptake value (SUV)_mean_ of brain regions of interest (ROIs) and the SUV_max_ of the cancer focus in colorectal cancer patients. After controlling for age, body mass index, and blood glucose, the partial correlation analysis showed that the SUV_mean_ of the left dorsolateral superior frontal gyrus (SFGdor.L) and the right SFGdor (SFGdor.R) was negatively associated with the SUV_max_ of the cancer focus.

After adjusting for age, BMI, and blood glucose, the partial correlation analysis showed a significant association between the SUV_max_ of the cancer focus and the SUV_mean_ of SFGdor.L (*r* = −0.418, *p* = 0.006) and SFGdor.R (*r* = −0.340, *p* = 0.028; Figure 6).

## Discussion

In this study, we implemented SSM/PCA and the metabolic connection between ROI pairs to determine cerebral metabolic patterns in CRC and chronic enteritis patients and analyze the relationships between the SUV_mean_ of the brain ROIs and the SUV_max_ of the cancer focus in CRC patients. The main findings of this study were as follows: (1) CRC and chronic enteritis patients exhibited characteristic cerebral metabolic patterns, which mainly involved areas responsible for visceral sensation and both affective and cognitive psychological processes; (2) the internal connectivity of the metabolic pattern in CRC patients was destroyed; and (3) in CRC patients, the SUV_max_ of the cancer focus was negatively correlated with the dorsolateral superior frontal gyrus. These findings indicated a characteristic cerebral metabolic pattern in the CRC population.

The study provided neuroimaging evidence for gut-brain crosstalk. Patients with CRC often experience abnormal defecation, abdominal distension, and abdominal pain. When such types of visceral stimulation are sensed, the enteric afferent nerve system (mainly the sympathetic and parasympathetic afferent systems) transfers signals from the gut to the brain (Skrobisz et al., 2019). The sympathetic afferent neurons project to the somatosensory cortex, insula, and other brain regions that produce the conscious visceral reflex and encode the intensity and location of visceral stimulation *via* the spinothalamic pathway (Dunckley et al., 2005). The prefrontal cortex then integrates and processes sensory and visceral pain signals. In contrast, the parasympathetic afferent neurons project to the solitary nucleus and then to the brain regions involved in the behavioral, neuroendocrine, and emotional autonomic responses *via* the thalamus (periventricular nucleus) (Price, 2000). The gut-brain axis is a bidirectional system of connections between the brain and the digestive tract. The brain’s extensive modulation of sensory signals stems from the intestines following both cognitive and affective psychological processes (Gregory et al., 2003). This is consistent with our finding that the characteristic cerebral metabolic pattern in CRC patients is mainly involved in visceral sensation and both affective and cognitive psychological processes.

More specifically, patients with CRC showed cerebral metabolic abnormalities in brain regions related to emotion and cognition, which included the frontoparietal, executive control, salience, and default mode networks. Several studies have shown that anxiety and depression occur in young and older adult patients with CRC (Agasi-Idenburg et al., 2017). It has also been reported that CRC patients experience a lack of positive emotions and fewer negative emotions than do those in the general population (Ciere et al., 2017). The close relationship between emotion and cognitive function has been demonstrated in numerous diseases. A large prospective study (Vardy et al., 2015) suggested that patients with CRC develop substantial long-term cognitive impairment with no significant added effect of chemotherapy. The cognitive domains affected were attention, working memory, verbal memory, and processing speed. Our findings provide neuroimaging evidence to support the above clinical studies and provide insight into the central mechanisms.

Perception is a conscious phenomenon that requires cortical processing (Ness and Gebhart, 1990). However, there is a fundamental difference between visceral and somatic sensation. The former is vague, whereas the latter is localized precisely to the site of origin. Studies have shown that cortical specialization in sensory-discriminative, affective, and cognitive areas of the cortex accounts for the perceptual differences observed between visceral and somatic sensation (Aziz et al., 2000). In our study, in addition to the brain regions related to both affective and cognitive psychological processes, we found cerebral metabolic abnormalities in brain regions related to visceral sensation, such as the insula, cingulate cortex, postcentral gyrus, paracentral lobule, and thalamus. This may be because visceral stimulation is more likely than somatic stimulation to induce intense autonomic and affective responses (Cervero, 1985). Visceral afferent information was transmitted to the thalamus along the spinal cord, and subsequently, sensory information was transmitted from the thalamus to the insular cortex and posterior central gyrus. The insular cortex is the region where visceral sensation is reprocessed and has been demonstrated in human and animal studies to be a crucial visceral sensory area (Augustine, 1996). In addition, numerous studies on visceral sensation have reported activation of the cingulate cortex during both painful and non-painful visceral stimulation (Aziz et al., 1997). We found metabolic abnormalities in the paracentral lobule and abnormal metabolic connections with the postcentral gyrus. Defecation disorder is a common symptom of CRC and is closely related to the paracentral lobule.

After identifying the cerebral metabolic pattern of CRC, we further extracted the SUV_max_ of the cancer focus and analyzed the partial correlations with the above ROIs. Although the correlation coefficient was small, we found that the SUV_max_ of the cancer focus was negatively correlated with the dorsolateral superior frontal gyrus, which is an essential region for cognitive functions (Huang et al., 2020), such as working memory, planning, inhibition, and abstract reasoning. Functional and structural abnormalities of the dorsolateral prefrontal cortex are often reported in studies of mental diseases (Peng et al., 2016), and cognitive impairment and emotional disorders are frequently exhibited by CRC patients. Our findings suggest a negative association between the severity of the disease and the function of the dorsolateral prefrontal lobe. Furthermore, a previous cancer-related study demonstrated that cancer patients show regional metabolic abnormalities in the prefrontal cortex, and the degree of disease roughly correlates with the degree of metabolic decline (Tashiro et al., 2003), which is consistent with our results.

We also studied patients with chronic enteritis to compare cerebral metabolic patterns between different intestinal diseases. We found that the metabolic patterns of patients with CRC and chronic enteritis were similar but not identical. Both showed metabolic abnormalities in brain regions related to visceral sensation and psychological processes. The involvement of the gut-brain axis in the pathogenesis of the two conditions may explain the similarity in brain metabolic patterns. The difference was mainly reflected in that patients with chronic enteritis were less than CRC in brain regions of visceral sensory and changes of metabolic connections between brain regions, which were due to the differences in the course and severity of the disease.

## Limitation

There were several limitations to this study. First, this study had a small sample size because of difficulties in recruiting CRC patients. Second, our study design was cross-sectional, and a longitudinal study will allow a better understanding of the correlation between cancer focus and brain regions. We plan to continue to acquire additional data for future studies.

## Conclusion

We found that the cerebral metabolic patterns of patients with CRC and chronic enteritis are related to visceral sensation and psychological processes, which is consistent with previous findings of clinical studies. Moreover, we revealed that the internal connections of the metabolic patterns in CRC patients had been destroyed. Our preliminary PET study revealed the neurobiological mechanisms underlying CRC and chronic enteritis and provides novel insight into gut-brain crosstalk.

## Data Availability Statement

The raw data supporting the conclusions of this article will be made available by the authors, without undue reservation.

## Ethics Statement

This retrospective study was approved by the institutional review board of Yueyang Hospital (No. 2020-188). Written informed consent for participation was not required for this study in accordance with the national legislation and the institutional requirements.

## Author Contributions

J-GX, X-YH, and M-XZ participated in the design of the study and data acquisition. JM drafted the manuscript. X-YH and M-XZ participated in the manuscript revision. J-JW, S-SL, and B-BH performed the statistical analysis. X-XX, Z-ZM, and XG carried out the data collection. All authors read and approved the final manuscript.

## Conflict of Interest

The authors declare that the research was conducted in the absence of any commercial or financial relationships that could be construed as a potential conflict of interest.

## Publisher’s Note

All claims expressed in this article are solely those of the authors and do not necessarily represent those of their affiliated organizations, or those of the publisher, the editors and the reviewers. Any product that may be evaluated in this article, or claim that may be made by its manufacturer, is not guaranteed or endorsed by the publisher.

## Footnotes

1
http://feinsteinneuroscience.org/imaging-software/download-software

2
http://www.fil.ion.ucl.ac.uk/spm/

## Abbreviations

BMI: body mass index
CRC: colorectal cancer
PCA: principal component analysis
ROI: region of interest
SSM: scaled sub-profile model
SUV: standard uptake value
SSF: subject scaling factor
VOIs: volume-of-interests.
